# Effect of Antihypertensive Therapy on SCORE-Estimated Total Cardiovascular Risk: Results from an Open-Label, Multinational Investigation—The POWER Survey

**DOI:** 10.1155/2013/165789

**Published:** 2013-07-25

**Authors:** Guy De Backer, Robert J. Petrella, Assen R. Goudev, Ghazi Ahmad Radaideh, Andrzej Rynkiewicz, Atul Pathak

**Affiliations:** ^1^Department of Public Health, Ghent University Hospital, DePintelaan 185, 9000 Ghent, Belgium; ^2^Department of Family Medicine and Cardiology, Lawson Health Research Institute, 801 Commissioners Road East, University of Western Ontario, ON, Canada N6C 5J1; ^3^Preventive Cardiology Clinic, Department of Internal Medicine, Medical University of Sofia, Sofia 1431, Bulgaria; ^4^Rashid Hospital, P.O. Box 4545, Oud Metha Road, Dubai, UAE; ^5^Department of Cardiology, Medical University of Gdañsk, M. Skłodowskiej-Curie 3a Street, 80-210 Gdañsk, Poland; ^6^Clinical Pharmacology Service, INSERM Unit 1048, Faculty of Medicine, University Hospital Toulouse, 31073 Toulouse, France

## Abstract

*Background.* High blood pressure is a substantial risk factor for cardiovascular disease. *Design & Methods.* The Physicians' Observational Work on patient Education according to their vascular Risk (POWER) survey was an open-label investigation of eprosartan-based therapy (EBT) for control of high blood pressure in primary care centers in 16 countries. A prespecified element of this research was appraisal of the impact of EBT on estimated 10-year risk of a fatal cardiovascular event as determined by the Systematic Coronary Risk Evaluation (SCORE) model. *Results.* SCORE estimates of CVD risk were obtained at baseline from 12,718 patients in 15 countries (6504 men) and from 9577 patients at 6 months. During EBT mean (±SD) systolic/diastolic blood pressures declined from 160.2 ± 13.7/94.1 ± 9.1 mmHg to 134.5 ± 11.2/81.4 ± 7.4 mmHg. This was accompanied by a 38% reduction in mean SCORE-estimated CVD risk and an improvement in SCORE risk classification of one category or more in 3506 patients (36.6%). *Conclusion.* Experience in POWER affirms that (a) effective pharmacological control of blood pressure is feasible in the primary care setting and is accompanied by a reduction in total CVD risk and (b) the SCORE instrument is effective in this setting for the monitoring of total CVD risk.

## 1. Introduction

Elevated blood pressure is a powerful contributor to total cardiovascular disease (CVD) risk. Clinical trials' experience supports the expectation of reduced cardiovascular risk from blood pressure control [1]. However, blood pressure control should not be applied in isolation. Rather, total CVD risk should be estimated in order to adapt the nature and intensity of preventive strategies to the circumstances of individual patients. A range of risk prediction models have been proposed for this purpose. One such is the Systematic Coronary Risk Evaluation (SCORE) model [2]. This instrument, designed for use in primary prevention, estimates the total risk of fatal CVD events over the next 10 years as a function of age, gender, systolic blood pressure (SBP), total cholesterol, and smoking habits.

Eprosartan (Abbott Products Operations AG, Allschwil, Switzerland) is an orally administered, nonpeptide, angiotensin-receptor blocker widely approved within the European Union and in the USA for the treatment of hypertension. The Physicians' Observational Work on patient Education according to their vascular Risk (POWER) study created opportunities to evaluate (a) the effect of treatment with eprosartan on SBP in a very large cohort derived from countries with varying degrees of total CVD risk (and different healthcare systems) and (b) the effect of eprosartan-based therapy (EBT) on total CVD risk as quantified by the SCORE model.

Patients were recruited from 16 countries: Bahrain, Belgium, Bulgaria, Canada, Croatia, Greece, Republic of Korea, Kuwait, Poland, Qatar, Russia, Saudi Arabia, South Africa, Sweden, Thailand, and UAE. Participants from 15 of these countries were evaluated for changes in their SCORE risk status during the treatment phase of POWER. Framingham risk equations were used to monitor changes in CVD risk status among patients in Canada.

We report here the principal findings of changes in SCORE risk distribution during this large international survey. Data from the Canadian contingent are the subject of a separate report.

## 2. Methods

The design and methodology of POWER have been described in detail in a separate publication [3]. In brief, POWER was an open-label, postmarketing surveillance study of 6 months' duration. Participating physicians (general practitioners or cardiologists) collected data for at least five sequentially recruited patients with (a) newly diagnosed mild-to-moderate hypertension (mean sitting SBP >140 mmHg) who physicians proposed to treat with eprosartan or (b) existing hypertension considered insufficiently well controlled by current therapy and in whom eprosartan could be safely added. 

The study protocol specified an initial regimen of eprosartan 600 mg/day. This could be supplemented with another antihypertensive agent (preferably hydrochlorothiazide (HCTZ) 12.5 mg/day) if the blood pressure response after 1 month of eprosartan monotherapy was considered insufficient. 

Patients were recruited from the 15 SCORE-eligible countries and stratified according to their country's standing in the SCORE risk distribution. These assignments were made after discussions with national SCORE coordinators. Canada was excluded from these arrangements because in that country the Framingham risk equation was used to monitor changes in CVD risk status.

### 2.1. Ethical Considerations

The protocol of the POWER study was developed in conformity with existent rules and guidance for good clinical practice and the ethical conduct of research in humans, including the precepts of informed consent, and was subject to Institutional Review Board and/or Ethics Committee review and approval as required by local regulations and practice. 

All patients were advised that they were free to withdraw from the study at any time and for any reason without prejudice to their subsequent medical care. 

Although initiated in advance of the publication of STROBE recommendations for the conduct of observational research, POWER was fully consistent with the provisions of that guidance.

### 2.2. Statistics

The primary objective of POWER was to assess the absolute change in SBP in a large hypertensive population treated with EBT for 6 months. 

Secondary efficacy variables included the absolute change in 10-year risk of fatal CVD assessed by SCORE from baseline to final visit. A chart-based SCORE estimate was generated centrally using the appropriate SCORE risk chart and individual patient data collected by physicians and recorded on case record forms. 

Nominal qualitative variables were compared using the Chi^2^ test, ordinal qualitative variables were compared using the Wilcoxon test, and quantitative variables were compared using analysis of variance. Descriptive statistics were prepared for safety data on all patients who received at least one dose of study treatment. 

## 3. Results

**Figure 1:**
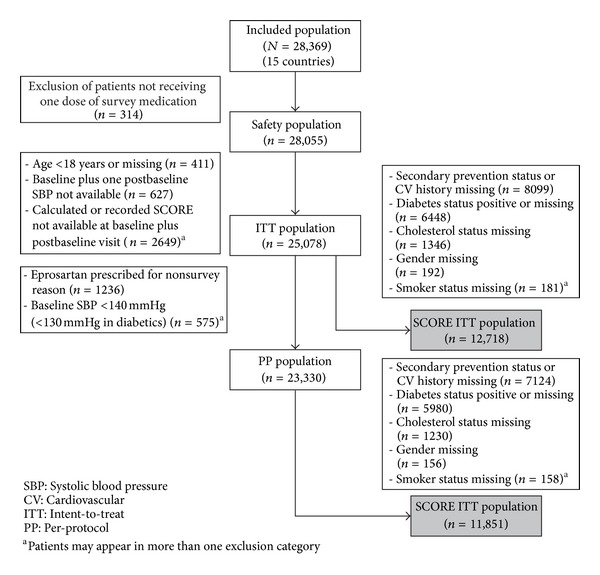
Patient disposition and derivation of the survey populations, including the SCORE ITT cohort.

Between May 2005 and October 2009 a total of 28,369 patients were recruited in 15 countries. The derivation of a safety population of 28,055 patients and an intent-to-treat (ITT) cohort of 25,078 patients is shown in Figure 1.

### 3.1. SCORE Population Data

After exclusion of patients in whom the SCORE risk estimation was not applicable (patients with established CVD or diabetes) and of patients recruited in Canada, there remained 12,718 patients who constituted a primary prevention population to which the SCORE methodology was applicable (Figure 1). This contingent had an almost 1 : 1 distribution of men (*n* = 6504) and women (*n* = 6214), although the women were on average older (61.2 ± 12 years versus 56.8 ± 12.1 years; *P* < 0.0001); a substantially greater proportion of women than men were 70 or more years old (27.1% (*n* = 1686) versus 15.4% (*n* = 1001); *P* < 0.0001 for overall age distribution). Overall, 26% of the patients were smokers; their mean cholesterol level was 5.50 ± 0.87 mmol/L, and 68.9% had a cholesterol level at baseline >5.1 mmol/L.

### 3.2. Blood Pressure Data

Baseline SBP/diastolic blood pressure (DBP) in the SCORE-eligible contingent was 160.2 ± 13.7/94.1 ± 9.1 mmHg; the intersex difference at baseline was <2 mmHg. Mean pulse pressure (PP) was 66.15 ± 14.01 mmHg, with a 2 mmHg higher average value in women than in men. PP also increased with age, due primarily to a trend towards a slight decrease in DBP with age. The distribution of hypertension categories in these patients (Table 1) was in conformity with the *≈*3 : 1 predominance of systodiastolic hypertension over isolated systolic hypertension seen in the overall ITT population [4]. 

Some 41.8% of patients in the SCORE ITT population were initially assigned to monotherapy (*n* = 5315); a further 33.0% (*n* = 4193) were assigned to dual therapy and 22.4% (*n* = 2847) were assigned to multidrug therapy (i.e., ≥3 drugs). Combination details were not recorded for 363 patients. The most often recorded drugs supplementing eprosartan at baseline were diuretics (including a fixed-dose combination of eprosartan with HCTZ; 27.4%; *n* = 3480), beta-blockers (26.5%; *n* = 3373), and calcium channel blockers (19.8%; *n* = 2519). Proportionately, more women than men were prescribed a combination of eprosartan plus a non-HCTZ diuretic (19.3% versus 12.6%). The percentage of patients in receipt of combination therapy increased with age (*P* < 0.0001 by Chi^2^ test).

The median duration of treatment was 182 days. During that time SBP and DBP declined by a mean of −25.6 ± 14.2 mmHg and −12.7 ± 9.2 mmHg, respectively, to a mean value of 134.5/81.4 mmHg. Mean PP fell by *≈*12.9 mmHg. A blood pressure response to therapy, defined as either SBP <140 mmHg and/or a reduction of SBP of ≥15 mmHg *or* DBP <90 mmHg and/or reduction of DBP of ≥10 mmHg, was recorded in 92.9% of patients; 62.9% of patients were classified as having normalized blood pressure at the end of observation, defined as SBP <140 mmHg and DBP <90 mmHg.

Significant absolute changes in other risk factors were also observed after 6 months, including a reduction in body mass index of −0.4 ± 1.2 kg/m² (*P* < 0.0001) and a 6.5% reduction in total cholesterol from 5.50 ± 0.87 mmol/L to 5.09 ± 0.74  mmol/L (*P* < 0.0001). Trends in the cholesterol distribution and smoker status during the study are shown in Table 2.

### 3.3. SCORE Data

The mean chart-based SCORE value was 6.0 ± 5.8% at baseline, with some sex difference (mean SCORE: men 7.6 ± 6.8%, women 4.4 ± 3.9%). The corresponding mean value on completion of observation was 3.5 ± 3.5% (men 4.4 ± 4.1%, women 2.5 ± 2.4%). The overall mean absolute reduction was −2.4 ± 3.1% (men −3.1%, women −1.7%); the overall mean relative reduction was −38.4% (men −38.4%, women −38.3%). Absolute risk increased with age at baseline (4.1 ± 3.4% at age 50–59 years, 10.9 ± 6.4% at ≥70 years) but the relative reduction was *≈*36% across all age groups.

**Figure 2:**
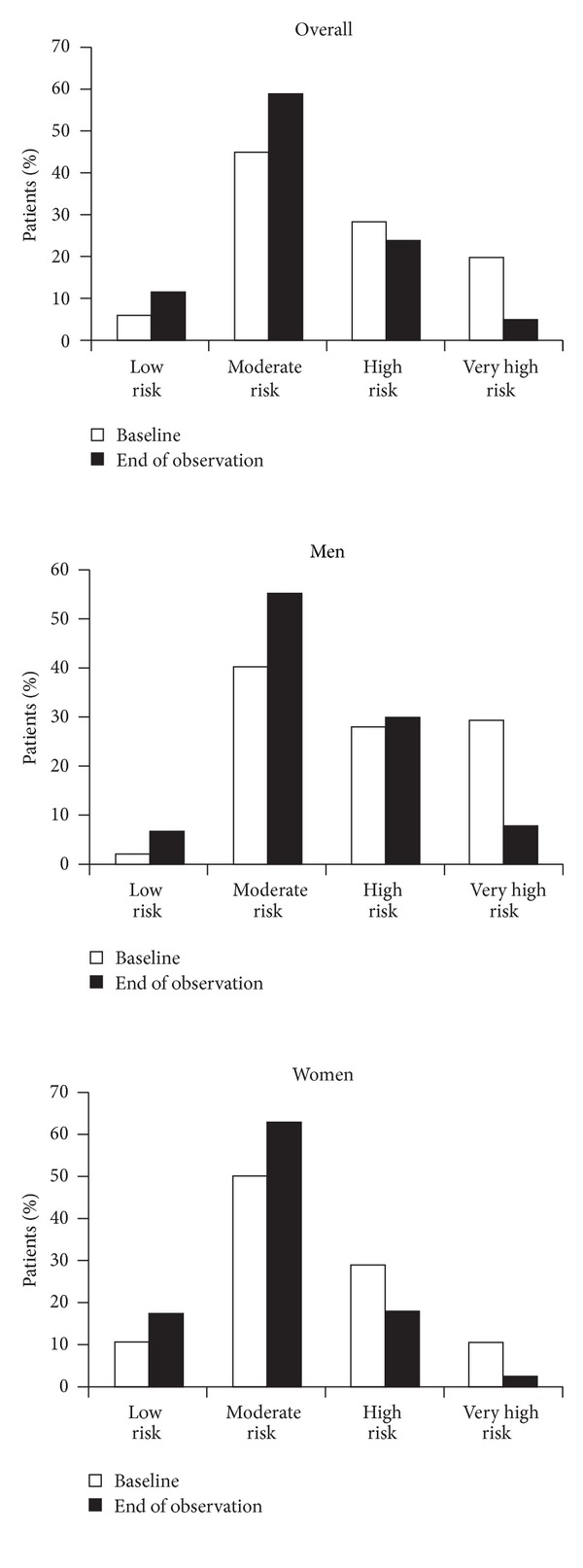
Chart-based estimates of SCORE risk distributions at baseline and at the end of observation, overall and by sex. Estimates based on *n* = 12,718 at baseline and *n* = 9577 at 6 months. Low risk <1%; moderate risk 1–4%; high risk 5–9%; very high risk ≥10%.

SCORE risk was stratified into four categories: low risk <1%; moderate risk 1–4%; high risk 5–9%; and very high risk ≥10%. The SCORE risk distribution among these patients at baseline and at the end of observation is depicted in Figure 2. At both recording points, there were marked sex differences within this overall finding: at baseline 10.6% of women were classified as low risk compared with 2.2% of men, whereas 29.9% of men were classified as very high risk compared with 10% of women; at the final visit (based on *n* = 9577), 17.1% of women were classified as low risk compared with 6.9% of men, whereas 8.1% of men were classified as being at very high risk compared with 1.9% of women (Figure 2).

**Figure 3:**
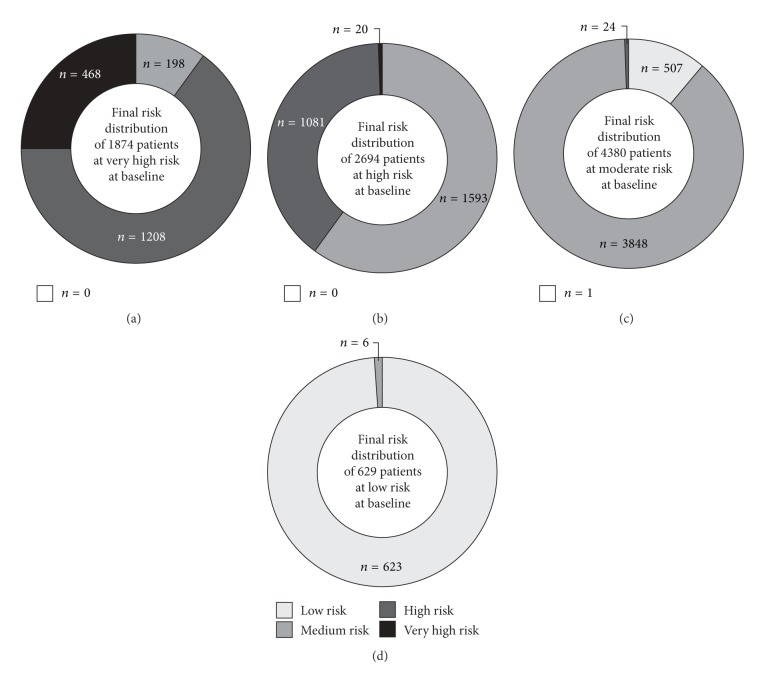
Shifts in SCORE risk distribution for the 9577 patients who generated chart-based estimates at baseline and at the conclusion of observation.

Figure 3 illustrates the shifts in risk distribution for the 9577 patients who provided chart-based SCORE estimates at baseline and at the conclusion of observation. 

Of the 1874 patients initially classified as being at very high risk, 75% were reclassified to lower-risk categories at the completion of observation (Figure 3).

Among patients initially classified as high risk, 59.1% were reclassified as either moderate or low risk, while 20 (*≈*0.7%) were reclassified as very high risk (Figure 3).

Most patients (87.9%) initially estimated to be at moderate risk remained in that category at the end of observation. A further 11.6% of patients were reclassified as low risk and 25 patients (<1%) were reclassified to higher-risk categories (Figure 3). 

Almost all patients (99%) initially classified as low risk were similarly classified at the completion of observation (Figure 3).

Overall, therefore, antihypertensive treatment for 6 months was associated with an improvement in SCORE risk of one category or more in 36.6% of patients (*n* = 3506), whereas 51 patients (*≈*0.5%) were considered to have experienced a deterioration of one category or more.

In analysis stratifying patients by age, the proportion of patients classified as low or moderate risk increased from 51.3% at baseline to 70.7% at the final clinical visit (data not shown in detail). At baseline, 8.4% of patients aged 70 years or more (*n* = 227) were classified as moderate risk, 43.7% (*n* = 1175) were classified as high risk, and 47.8% (*n* = 1285) as very high risk. At the completion of the observation phase, these proportions had shifted to 28.3% (*n* = 529), 57.9% (*n* = 1082), and 13.9% (*n* = 259), respectively. 

An exploratory analysis comparing the CVD risk profile of the 9577 patients for whom SCORE data were available at baseline and at 6 months with the 3141 patients who had SCORE data available only at baseline identified no clinically relevant differences that might have influenced the SCORE findings (data not shown). 

Among patients whose overall SCORE risk improved by at least one category, 97% had a reduction in SBP of at least one grade, 37% achieved an improvement of at least one grade in cholesterol status, and 6% reported stopping smoking during the study.

### 3.4. Safety Findings

Safety data were accrued from a population of 28,055 patients recruited in the countries that used SCORE methodology to calculate CVD risk (Figure 1). Within that population, a total of 374 events (in 298 patients) were classified as suspected adverse drug reactions (SADRs). Summary details of these SADRs, and of in-study deaths, appear in Table 3. 

Two of the five deaths recorded were classified as SADRs. Causes of death in these cases comprised coma and cerebral bleeding. The deaths recorded as not being an SADR were attributed to lung embolism and ovarian cancer, unexpected death, and cerebrovascular accident in one patient each. The deaths associated with coma or cerebral bleeding were formally classified as suspect because a possible causal relation to use of study medication was not indicated by the investigators. 

## 4. Discussion

In this open-label observational intervention of 6 months' duration, antihypertensive therapy based on eprosartan (with additional agents as considered necessary) was associated with a mean −25.6 ± 14.2 mmHg reduction in SBP in a population with predominantly systodiastolic hypertension. Mean DBP and PP both fell by more than 10 mmHg. These reductions, especially in SBP, may be larger than what might have been reported in a controlled study but are in keeping with the high degree of innate variability in blood pressure. As an open-label, observational exercise, POWER does not offer the rigor of a controlled trial. However, it conforms to the provisions of the SCOPE principles for open-label research [5], and the wide geographical spread of participating countries provides some assurance against the operation of systematic biases with potential to distort the results.

Given the established relation between systemic arterial blood pressure and CVD risk, it might be expected that the blood pressure reductions observed in this study would be accompanied by improved overall CVD risk status, and this was indeed the case. Among >9000 patients with no baseline diagnosis of CVD or diabetes mellitus (i.e., a true primary prevention population), the period of EBT was characterized by improved SCORE-estimated risk status in 36.6% of the patients. Among 1874 patients initially classified as being at very high CVD risk (defined as 10-year risk ≥10%), 1406 (75%) achieved improvements in SCORE status of at least one category by the end of the observation phase (Figure 3). Similarly, 1593 of 2694 patients (59.1%) initially classified as being at high risk achieved improvements in SCORE status of at least one division by the end of the observation phase (Figure 3). Fewer than 1% of participants (*n* = 51) experienced a deterioration in SCORE risk status during the period of observation, a finding that may be seen as further evidence of a cause-effect relation between the reduction in blood pressure and the improvement in CVD risk status. 

Reference to the SCORE risk charts (available at http://www.escardio.org/communities/EACPR/toolbox/health-professionals/Pages/SCORE-Risk-Charts.aspx#countries) reveals that for nonsmoking patients similar to ours (age *≈*60 years, SBP *≈*160 mmHg, baseline total cholesterol *≈*5 mmol/L), relative risk varies several-fold and that an approximately 25 mmHg reduction in SBP, as was observed in POWER, can be expected to bring the relative risk of those initially in the higher-risk categories much closer to that of their peers, who are at low relative risk. In women—although less so in men—absolute 10-year risk may be brought to very low levels, as was the case in our patients. 

Relative risk status may be further modified by smoking cessation. Physicians participating in POWER were mandated to counsel patients about smoking cessation but no standard instruments or programs were deployed for that purpose. Our data indicate that some 5% of patients reported smoking cessation during POWER. It may reasonably be assumed that few, if any, patients who stopped smoking did not report that fact. Hence, it seems likely that in this population smoking cessation played only a small part in the reductions in CVD risk status recorded via SCORE. As smoking cessation may be expected to halve the relative risk in patients such as ours and moderate the 10-year risk of a CVD event, the case for the addition of a smoking cessation program to control blood pressure as part of routine practice seems compelling. The work of Rodondi et al. offers one interesting perspective on this aspect of CVD risk management [6].

Inadequate control of hypertension is a major contributor to excess CVD mortality [7]. Our data reaffirm that effective pharmacological control of blood pressure is feasible in the primary care setting. It is thus a matter for concern that reports of widespread inadequate hypertension management persist in the medical literature [8–10]. We concur with Zannad et al. [11] that risk scoring systems such as SCORE have limitations but also agree that they provide a solid and accessible starting point for preventive cardiology involving minimum cost and complexity, and with no meaningful barriers to use. Given their relative ease of use, the SCORE charts are a practical resource for general practice. They are also to be preferred to unstructured physician estimates of risk [12].

Various proposals have been advanced for refining the accuracy and dependability of the SCORE technique [13–15]. However, application of the original SCORE charts appears to produce satisfactorily accurate risk estimates [16], and it seems to us that a much more significant impact on population CVD risk will be obtained from promoting a consistent and systematic use of the basic SCORE instrument (or similar) than from further attempts to refine the instrument without parallel efforts to encourage its use. Even then, the full value of using SCORE is likely to be realized only when it is integrated into a structured program of preventive activities. Experience from initiatives such as the Education and Coronary Risk Evaluation (EDUCORE) [17] and INterventions for COntrol of hyperTEnsion in CAtalonia (INCOTECA) [18] may prove instructive in shaping the delivery of primary preventive care. The need for continuing physician education also needs to be acknowledged [19].

## 5. Conclusions

In this open-label observational study, the use of EBT was associated with reduction in systemic blood pressure and associated CVD risk. The SCORE instrument was confirmed as an effective method for estimating and monitoring CVD risk in primary care. 

## Figures and Tables

**Table 1:** Hypertension classification in the SCORE-eligible contingent of POWER (*n* = 12,718).

Patient population	Male (*n* = 6504)	Female (*n* = 6214)	Total population (*n* = 12,718)
Patients contributing data	6477	6172	12,649
Isolated systolic hypertension; *n* (%)	1296 (20.0)	1515 (24.5)	2811 (22.2)
Isolated diastolic hypertension; *n* (%)	92 (1.4)	58 (0.9)	150 (1.2)
Systodiastolic hypertension; *n* (%)	5026 (77.6)	4529 (73.4)	9555 (75.5)
No hypertension (SBP <140 mmHg and DBP <90 mmHg); *n* (%)	63 (1.0)	70 (1.1)	133 (1.1)
Missing values	27	42	69

SBP: systolic blood pressure; DBP: diastolic blood pressure.

**Table 2:** Trends in total cholesterol distribution and smoking status during the POWER study.

	At baseline (*n* = 12,718)	At final visit (*n* = 9909)
	Cholesterol distribution; *n* (%)	
≤4.5 mmol/L	1602 (12.6)	2067 (20.9)
4.5–5.1 mmol/L	2351 (18.5)	2777 (28)
>5.1 mmol/L	8765 (68.9)	5065 (51.1)

	Smokers (%)	
Smoking status: yes	26	23.3

**Table 3:** Summary of suspected adverse drug reactions (SADRs) and in-study deaths recorded during POWER.

Adverse event	Number of events (No. of patients)
SADRs	374 (298)
SADRs leading to study discontinuation	255 (205)
Serious SADRs	14 (11)
Severe SADRs	36 (29)
Deaths	5 (5)

## References

[1] Turnbull F (2008). Effects of different regimens to lower blood pressure on major cardiovascular events in older and younger people: meta-analysis of randomised trials. *British Medical Journal*.

[2] Conroy RM, Pyörälä K, Fitzgerald AP (2003). Estimation of ten-year risk of fatal cardiovascular disease in Europe: the SCORE project. *European Heart Journal*.

[3] De Backer G, Petrella RJ, Goudev AR, Radaideh GA, Rynkiewicz A, Pathak A (2013). Design and methodology of POWER, an open-label observation of the effect of primary care interventions on total cardiovascular risk in patients with hypertension. *Fundamental and Clinical Pharmacology*.

[4] Goudev A, Berrou J-P, Pathak A (2012). Effect of eprosartan-based therapy on systolic blood pressure and total cardiovascular risk in a large international population: preliminary report of the POWER observational study. *Vascular Health and Risk Management*.

[5] Von Elm E, Altman DG, Egger M, Pocock SJ, Gøtzsche PC, Vandenbroucke JP (2007). The Strengthening the Reporting of Observational Studies in Epidemiology (STROBE) statement: guidelines for reporting observational studies. *PLoS Medicine*.

[6] Rodondi N, Collet T-H, Nanchen D (2012). Impact of carotid plaque screening on smoking cessation and other cardiovascular risk factors: a randomized controlled trial. *Archives of Internal Medicine*.

[7] Guallar E, Banegas JR, Blasco-Colmenares E (2011). Excess risk attributable to traditional cardiovascular risk factors in clinical practice settings across Europe—The EURIKA Study. *BMC Public Health*.

[8] Centers for Disease Control and Prevention (CDC) (2012). Vital signs: awareness and treatment of uncontrolled hypertension among adults—United States, 2003–2010. *Morbidity and Mortality Weekly Report*.

[9] Banegas JR, López-García E, Dallongeville J (2011). Achievement of treatment goals for primary prevention of cardiovascular disease in clinical practice across Europe: the EURIKA study. *European Heart Journal*.

[10] Missault L, Witters N, Imschoot J (2010). High cardiovascular risk and poor adherence to guidelines in 11 069 patients of middle age and older in primary care centres. *European Journal of Cardiovascular Prevention and Rehabilitation*.

[11] Zannad F, De Backer G, Graham I (2012). Risk stratification in cardiovascular disease primary prevention—scoring systems, novel markers, and imaging techniques. *Fundamental and Clinical Pharmacology*.

[12] Bruckert É, Bonnelye G, Thomas-Delecourt F, André L, Delaage P-H (2011). Assessment of cardiovascular risk in primary care patients in France Évaluation du risque cardiovasculaire en médecine générale en France. *Archives of Cardiovascular Diseases*.

[13] Merry AH, Boer JM, Schouten LJ (2012). Risk prediction of incident coronary heart disease in the Netherlands: re-estimation and improvement of the SCORE risk function. *European Journal of Cardiovascular Prevention & Rehabilitation*.

[14] Sehestedt T, Jeppesen J, Hansen TW (2010). Risk prediction is improved by adding markers of subclinical organ damage to SCORE. *European Heart Journal*.

[15] Wierzbicka-Chmiel J, Mizia-Stec K, Haberka M, Chmiel A, Mizia M, Gasior Z (2009). The relationship between cardiovascular risk estimated by use of SCORE system and intima media thickness and flow mediated dilatation in a low risk population. *Cardiology Journal*.

[16] De Bacquer D, De Backer G (2010). Predictive ability of the SCORE Belgium risk chart for cardiovascular mortality. *International Journal of Cardiology*.

[17] Rodríguez-Salceda I, Escortell-Mayor E, Rico-Blázquez M (2010). EDUCORE project: a clinical trial, randomised by clusters, to assess the effect of a visual learning method on blood pressure control in the primary healthcare setting. *BMC Public Health*.

[18] Vallès-Fernandez R, Rosell-Murphy M, Correcher-Aventin O (2009). A quality improvement plan for hypertension control: the INCOTECA Project (Interventions for Control of hypertension in Catalonia). *BMC Public Health*.

[19] Shaikh RB, Mathew E, Sreedharan J, Muttappallymyalil J, Sharbatti SA, Basha SA (2011). Knowledge regarding risk factors of hypertension among entry year students of a medical university. *Journal of Family and Community Medicine*.

